# Migration Ecology and Protection of Stopover Sites of the Whimbrels Along China's Coastal

**DOI:** 10.1002/ece3.71890

**Published:** 2025-08-08

**Authors:** Ke He, Zhen Xian Zhu, Tinghao Jin, Jun Yan Feng, Xilai Zhou, Lingling Wei, Baoquan Liu

**Affiliations:** ^1^ College of Animal Science and Technology College of Veterinary Medicine, Zhejiang Agriculture and Forestry University, Key Laboratory of Applied Technology on Green‐Eco‐Healthy Animal Husbandry of Zhejiang Province Hangzhou China; ^2^ Zhejiang Forest Resource Monitoring Center Hangzhou Zhejiang China

**Keywords:** habitat protection, migration network, migration of shorebirds, satellite tracking, stopover sites

## Abstract

To effectively establish a conservation network for migratory bird flyways in China, understanding the protection status of the East Asian‐Australasian Flyway (EAAF) along China's coastal regions is a prerequisite. Using satellite tracking data from 13 Whimbrels ( *Numenius phaeopus* ) in Hangzhou Bay (2018–2023), we found (1) After traversing the coastal and the Northeast Plain in China, the Whimbrels captured in Hangzhou Bay diverged into two distinct migration routes at 45°N–50° N. There was no difference in migration parameters between individuals on different routes, except for the speed of southward migration (*p* < 0.05). (2) The Hangzhou Bay tracked individuals exhibited strong fidelity to this wetland and had a high degree of overlap in the concentrated activity zones there. (3) Combined with other published studies, we evaluated the protection gaps, revealing 73.1% (38 of 52) stopover sites fall within protected areas (primarily national‐level), but with stark regional disparities: inland regions (Inner Mongolia and Northeast China) showed lower protection coverage than coastal zones (East Sea‐South Sea and the Bohai Sea‐Yellow Sea of China). These integrated findings demonstrate that while China's coastal protected areas effectively cover most key Whimbrel habitats, critical inland stopovers remain unprotected. We recommend a need for greater conservation attention in inland regions. The findings of this study provide a theoretical foundation for effectively establishing a migratory bird flyway conservation network in China.

## Graphical abstract

Our analysis reveals two critical findings: (1) 73.1% of identified stopover sites occur within existing protected areas, demonstrating significant conservation coverage; yet (2) substantial protection gaps persist, particularly in inland regions of Inner Mongolia and Northeast China where 42.9% of key sites lack formal protection.

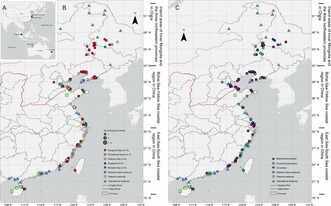

## 1 Introduction

Many shorebirds undertake annual long‐distance migrations between breeding and non‐breeding regions. Their populations are influenced by multiple factors, including climatic conditions, food availability, predation pressure, and habitat quality (Rosenberg et al. 2019; Studds et al. 2017). Evidence from multiple studies indicates a declining trend in some migratory bird populations (Bairlein 2016; Kirkland et al. 2025). Twenty‐six of 28 (93%) shorebird species are decreasing in the Americas (1980–2019) (Smith et al. 2023) and 12 of 42 (29%) wintering waterbird species are declining in East Asia (1998–2017, Deep Bay, South China) (Sung et al. 2021), while populations of Afro‐Palearctic migrant birds have shown a pattern of sustained, often severe, decline (Fiona et al. 2006). Stopover sites enable migratory birds to replenish energy reserves, recover physiologically, and seek refuge from harsh weather while serving as critical connectors between breeding and wintering grounds (Horton and Deng 2024). Consequently, this role makes their conservation absolutely critical for sustaining migratory bird populations.

Coastal wetlands in China, situated within the East Asia‐Australasia Flyway (EAAF), serve as vital stopover sites for migratory birds, owing to their extensive area and abundant wetland resources. Chinese “Action Plan for the Protection and Restoration of Migratory Bird Flyways (2024–2030)” aims to bring 90% of critical habitats within migratory bird flyways under effective protection by 2030, establishing a comprehensive national protection network for migratory bird flyways.

Based on synchronized waterbird surveys in China, several coastal wetlands have been designated as priority conservation areas (Bai et al. 2015; Conklin et al. 2014; Xia et al. 2017). Notably, the Bohai Sea‐Yellow Sea wetlands host at least 36 shorebirds, with a combined population exceeding two million individuals. Among these, 18 species represent over 30% of their global migratory populations (Duan et al. 2019; Aharon‐Rotman et al. 2016). In this area, key stopover sites for shorebirds include the Yangtze River Estuary, Yancheng in Jiangsu, the Yellow River Delta, northern Bohai Bay, the Shuangtai River Estuary, and the Yalu River Estuary (Conklin et al. 2014; Duan et al. 2019; Xia et al. 2017). Besides the regions mentioned above, the Hangzhou Bay coastal wetland on China's southeast coast ranks among the most productive wintering habitats for waterbirds, mainly owing to its advantageous geographical location (Gao et al. 2024). The long‐term monitoring (Gao et al. 2024) and bird ringing (Liu et al. 2024) both underscore Hangzhou Bay's dual significance as a crucial wintering ground and a major stopover site for shorebirds, supporting large and stable populations.

Among these species, the Whimbrel is a pivotal subject for studying the migration patterns of shorebirds. As a long‐distance migrant, the EAAF hosts approximately 65,000 Whimbrels (Hansen et al. 2016). Research reveals that the initial flight from its non‐breeding regions (Moreton Bay and Roebuck Bay in Australia) accounts for over half of the total spring migration distance, and the time spent at stopover sites during spring and autumn migrations constitutes more than half of the entire migration duration (Kuang et al. 2020), emphasizing the critical role of stopover sites in energy replenishment for this species. The Whimbrel demonstrates a highly consistent migratory behavior, particularly during northward migrations. Individual birds repeatedly utilize stopover sites along the Yellow Sea coast over multiple years, and their residence time at these frequently visited sites is significantly longer than that at those used only once (An et al. 2024). Its high detection frequency and ringing recapture rate during the waterbird‐ringing efforts in Hangzhou Bay also underscore its significance. In this study, we used the tracking data of Whimbrel to analyze the migration routes and stopover sites used by individuals captured at the stopover site Hangzhou Bay during their northward migration. The protection levels of crucial stopover sites within shorebirds migratory corridors were also analyzed and discussed, integrated with tracking data from other ringed Whimbrels. This research provides a scientific foundation for formulating effective protection strategies for shorebirds migrations.

## 2 Individuals and Methods

### 2.1 Studied Individuals

The studied Whimbrels were captured during bird‐ringing activities in Hangzhou Bay in spring (April and May) from 2018 to 2023. These birds were caught using mist nets on the intertidal mudflats, fitted with metal rings and trackers before release. Hangzhou Bay (29°58′27″‐30°51′30″ N, 120°54′30″‐121°50′48″ E) is a typical trumpet‐shaped, strong‐tidal estuary. Its northern shore features an eroded coastline, while the southern shore is characterized by silt‐filled mudflat wetlands. Long‐term monitoring in this region reveals that during the wintering period, this region supported more than 20,000 individuals per year, and five shorebirds—Dunlin ( *Calidris alpina* ), Kentish Plover ( *Charadrius alexandrinus* ), Gray Plover ( *Pluvialis squatarola* ), Pied Avocet ( *Recurvirostra avosetta* ), and Curlew ( *Numenius arquata* )—consistently meet the 1% population level Ramsar listing criterion at least once at one site for the EAAF (Gao et al. 2024). Analysis of bird‐ringing recapture records (2018‐present) suggested waders showing strong site fidelity (Liu et al. 2024). These findings underscore Hangzhou Bay's dual significance as both a crucial wintering ground and a major stopover site for shorebirds, supporting large and stable populations.

### 2.2 Tracking Devices

The trackers used were backpack‐style, made of nylon, and would not fall off automatically (100 tracker‐06, produced by Hangzhou Track Technology Company). Each machine weighed 6 g and measured 26 mm × 22 mm × 13 mm, accounting for 1.63%–2.24% of the body weight of the ringed individuals. This weight complied with the standard that tracker weight should be less than 3% of the wild animal's body weight. The device was set to return information every 8 h, and the collected information was transmitted via the Global System for Mobile Communications (GSM) and received through the Chinese Mobile Communication System. Satellite positioning data were downloaded from a web client after decryption.

### 2.3 Data Analysis

Thirty‐eight adult individuals had tracking devices attached; data were retrieved by downloading tracking information from the tracker's web client. After removing invalid location data (abnormal location coordinates, e.g., values like 999 or 0), the remaining satellite‐derived coordinates were imported into ArcGISPro 3.1.5. These coordinates were then sequentially connected based on time stamps to visualize migration routes. We defined “N1” as the spring migration from Hangzhou Bay to breeding regions, “S” as the autumn migration (Southward) from breeding regions to non‐breeding regions, and “N2” as the spring migration from non‐breeding regions to Hangzhou Bay. “Northward” encompassed both N1 and N2. Based on the results, individuals that did not have at least two periods (N1‐S) were not included in this study, and the remaining individuals were 13 (Table S1). Individual L103 showed repeated N‐S phases (N1‐S‐N2‐N1′‐S′), which we split into two separate migration cycles: L103‐1 (N1‐S‐N2, 2019–2020) and L103‐2 (N1′‐S′, 2020–2021) to avoid pseudo‐replication. Thus, we had 14 migration data.

#### 2.3.1 Divergence Between Groups Following Different Migration Routes

Five subspecies have been recognized in the Whimbrel in Eurasia, and Kuang et al. (2022) suggest that both ssp. *rogachevae* and ssp. *variegatus* occur in the EAAF. Tracked individuals were classified into distinct migratory route populations according to the longitude and latitude of breeding regions (Kuang et al. 2022): (1) the Western migratory population, breeding within the longitude range of 100°–125° E; (2) the Eastern migratory population, breeding at longitudes between 130°–150° E; (3) the Kamchatka Peninsula migratory population, which traverses the Kamchatka Peninsula and breeds in the 160°–180° E region.

#### 2.3.2 Migratory Route Analysis

The analyzed data included migration duration, distance, speed, flight speed, stopover sites, number of stopover sites, and stopover duration. Given that all study individuals were captured in Hangzhou Bay, the N1 duration specifically denoted the time from departing Hangzhou Bay to reaching the breeding region, encompassing stopover periods. Migration speed was calculated as total distance divided by duration. Flight time excluded stopover periods from the migration duration, with flight speed derived by dividing migration distance by this adjusted flight time. Stopover sites were identified based on satellite tracker‐recorded position data: an individual was deemed to have stopped if its activity was confined within an area spanning 50 km or 0.5° latitude (Kuang et al. 2022). Stopovers were classified according to the duration of stay, with periods exceeding 48 h considered stopover events (Kuang et al. 2022). The first location within this stationary period was recorded as arrival time, and the last location before moving beyond 50 km was recorded as departure time. Stopover duration was calculated from these timestamps. At these stopover sites, individuals were assumed to forage, replenish resources, and accumulate energy for subsequent migration phases. For non‐breeding areas, all movements south of Southeast Asia were classified as non‐breeding regions.

To assess the consistency of migration routes, a spline interpolation function was employed to estimate the longitudes of individuals traversing specific latitudes. For the N1 migration, latitudes from 35° to –65° N were set at 5° intervals (7 latitude points in total). For the S migration, 10 latitude points were set from 10° to –60° N at 5° intervals, and for the N2 migration, 5 latitude points were set from 10° to –30° N at 5° intervals. All analyses were conducted using the Geosphere package in R software (v 4.4.1). Due to unstable tracker signals at breeding areas and missing data on individuals departing these areas, the average departure time of individuals with similar migration paths were used as references: 30th July for the Eastern migratory population and 2nd August for the Western migratory population (Kuang et al. 2022). The Mann–Whitney test was performed to compare migration parameters across different breeding populations.

#### 2.3.3 Analysis of Stopover Sites

We performed a multi‐scale assessment of stopover site status. Firstly, to investigate the impact of latitude on the stopover duration at stopover sites, we aggregated the stopover sites of various individuals within the transit range of the Hangzhou Bay population. Then, we extracted the average latitude of these stopover sites as the independent variable and the stopover time as the dependent variable. These variables were analyzed using a Generalized Additive Model (GAM), implemented via the mgcv package in R software. For the GAM, we employed thin‐plate regression splines to define smoothing terms and used the Restricted Maximum Likelihood (REML) method for parameter estimation. To effectively capture the nonlinear relationships in the data, the maximum degree of freedom for the smoothing term was set at 10.

Second, we partitioned the whole stopover sites into five latitude‐based regions: the Southeast Asian region, the East Sea‐South Sea coastal region in China, the Bohai Sea‐Yellow Sea coastal region in China, the inland areas of Inner Mongolia and the three northeastern provinces in China, and the Siberian region. Three of them are in China. The demarcation between the East Sea‐South Sea coastal region and the Bohai Sea‐Yellow Sea coastal region was set at the northern shore of the Yangtze River estuary. We compared the mean number of stopover times, mean total stopover duration, and single mean stopover duration across these regions. Across all 13 tracked individuals combined, we recorded 58 stopover events during the N1 phase, 31 during the N2 phase, and 10 during the S phase. For the East Sea‐South Sea coastal area, which had data from both N1 and N2 phases, we calculated values separately using the respective monitoring phase counts and then obtained an average. For stopover sites utilized multiple times, we analyzed the activities within these areas according to different migration stages (N and S). To represent the stopover activity range in some reused stopover sites, we employed Kernel density analysis (estimated at 95% density, using the default parameters) and used ArcGIS to compute the area and map the activity regions.

Thirdly, we gathered published data on the stopover status of Whimbrel in Chinese coastal regions, which were the East Sea–South Sea coastal region in China, the Bohai Sea–Yellow Sea coastal region in China, the inland areas of Inner Mongolia, and the three northeastern provinces in China in previous studies, to discuss the stopover site fidelity of individuals from different ringing sites (Kuang et al. 2020; Kuang et al. 2022). Individual data from the ringing sites of Moreton Bay (MB), Australia, Roebuck Bay (RB), Australia, Singapore, and Chongming Dongtan (CMDT) were incorporated. At the population level, we merged individual stopover sites within 25 km (half of the stopover range) of each other to calculate central locations through coordinate averaging. This 25‐km threshold ensured that any two merged individuals had at least 50% overlap in their potential activity ranges. We superimposed the distribution of Chinese national nature reserves, international wetland lists, national wetland parks, national forest parks, and provincial‐level nature reserves on the stopover site distribution. We calculated the average latitude and longitude of the stopover sites (by individuals) and their distances from these protected areas and used 25 km (half of the stopover range) as a criterion to determine whether a stopover site was within a protected area. We considered a stopover site as protected if its mean location was within 25 km of a protected area boundary since this buffer (half our 50‐km stopover radius) ensures substantial overlap with actual protected zones. The stopover situations were classified into three levels: national‐level protection (within national nature reserves, national wetland parks, and national forest parks), provincial‐level protection (within provincial‐level nature reserves), and unprotected. The first two levels were considered protected. If a site met the criteria for both national‐ and provincial‐level protection, it was defined as having national protection. Subsequently, we evaluated the protection status of the migration corridors of the Whimbrel. Differences in stopover duration across protection levels were analyzed using the Mann–Whitney U test.

## 3 Result

### 3.1 Analysis of Individual Migration Routes From Hangzhou Bay

**FIGURE 1:**
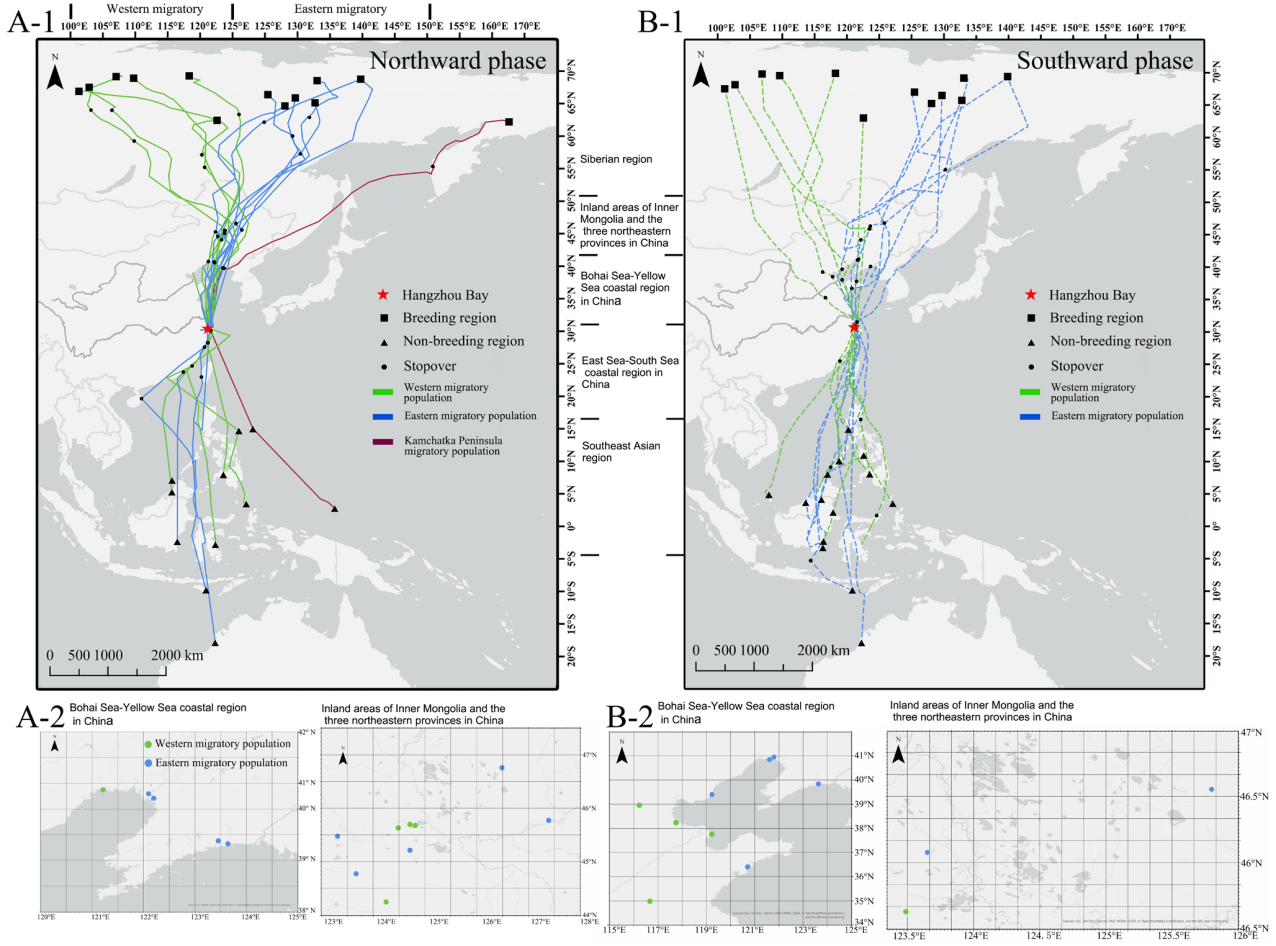
Migration routes of Whimbrels in this study, using the WGS84 Coordinate System. (A‐1) The Northward phase route; (A‐2) enlarge of stopover sites within China after leaving Hangzhou Bay; (B‐1) the Southward phase route; and (B‐2) enlarge of stopover sites within China before arriving at Hangzhou Bay. Notably, individual B123 uniquely used two breeding sites, likely due to challenges at the first location.

A total of 10,136 satellite locations were obtained from the 13 tracked individuals, with a tracking duration of 408.5 ± 193.4 days (Figure 1, Table 1). During the N1 northward migration, the Whimbrel departed from Hangzhou Bay on 16th May (ranging from 9th May to 27th May) and reached the breeding regions on 2nd June (ranging from 26th May to 11th June). The migration distance was 4888 ± 536 km (ranging from 3919 to 5803 km), the migration time was 16.5 ± 4.4 days (ranging from 9.6–25.3 days), and they had 2–4 stopover sites with a total stopover duration of 27.7 ± 6.0 days (ranging from 14.3 to 36.5 days). For the southward migration (S) starting from the breeding site, on average, it began on 2nd August (ranging from 30th July to 14th August, no difference between two migration strategies). Ten individuals (76.92%) used Hangzhou Bay as a stopover site again. The non‐breeding areas included the Southeast Asian region, Pacific Ocean islands, and northwestern Australia, showing a wide latitudinal distribution. In the following year, they left the non‐breeding area on 23rd April (ranging from 15th April‐2nd May) to initiate the N2 stage (*n* = 5). Due to the limited recorded sample size and high variability in migration routes (only 3 individuals reused Hangzhou Bay as a stopover, 1 diverged, and 1 lost signal before arrival), N2‐stage data were excluded from the analysis but were provided in Table 1 for reference.

**TABLE 1:** The migratory parameters of Whimbrel in this study.

Parameters	Period	Eastern migratory population (*n* = 7)		Western migratory population (*n* = 6)		Kamchatka Peninsula population (*n* = 1)	All individuals (*n* = 13)	
		Mean	SD	Mean	SD	Value	Mean	SD
Distance	N1	4854	493	4776	521	5803	4888	536
	S	8550	1686	7890	866	No data	8245	1361
	N2	3995	1527	3749	381	No data	3897	1104
Duration time of migration	N1	16.0	5.1	15.9	2.8	23.4	16.5	4.3
	S	56.6	11.4	49.4	8.0	37.3	52.1	10.8
	N2	10.5	2.2	9.2	6.6	No data	10.0	3.7
Duration time of flight	N1	7.5	2.5	8.9	4.2	6.1	8.0	3.2
	S	18.6	8.0	9.4	3.5	37.3	16.5	9.8
	N2	9.4	0.3	4.5	0.0	No data	7.4	2.7
Migration speed (km/day)	N1	718.2	239.8	618.3	226.2	953.5	314.0	82.7
	S	157.2	24.7	165.7	44.0	No data	161.2	33.6
	N2	378.1	120.1	532.2	341.5	No data	439.7	208.5
Flight speed (km/day)	N1	329.8	103.5	306.6	60.3	247.6	692.2	233.2
	S	539.7	278.5	955.7	279.8	No data	713.1	341.6
	N2	426.4	160.5	829.7	79.2	No data	587.7	251.5
No of stopover sites	N1	2.4	0.5	2.5	0.8	3.0	2.5	0.7
	S	2.7	1.3	2.0	0.6	No data	2.4	1.0
	N2	1.7	0.6	2.0	0.0	0.0	1.6	0.7
Duration of stopovers (days)	N1	24.7	6.2	30.7	4.7	30.5	27.7	6.0
	S	37.8	12.5	32.6	14.5	No data	35.4	13.2
	N2	11.7	5.7	19.7	10.4	No data	16.3	9.1
Time leave Hangzhou Bay^a^		45.6	6.6	46.7	6.1	38.0	45.5	6.2
Time arrival at breeding region		61.7	5.2	63.7	5.2	62.0	62.6	4.9
Time leave breeding region		124.3	5.8	122.6	1.5	121.0	123.4	4.3
Time arrival at wintering region		180.9	16.5	171.5	6.4	158.0	175.2	13.7
Time leave wintering region		392.0	4.0	382.0	4.4	No data	387.0	6.6

^a^ The temporal data were calculated using April 1st as Day 1. The information of Kamchatka Peninsula individual's S stage.

### 3.2 Comparison of Individuals With Different Migration Directions

Based on the longitude of the breeding area (Kuang et al. 2022), a total of six Western migratory individuals, seven Eastern migratory individuals, and one individual belonging to the Kamchatka Peninsula were identified for the individuals ringed in Hangzhou Bay (Table S1; Figure 1). Due to the longitudinal difference between the Western and Eastern migratory populations in the breeding area, we analyzed the longitudinal variation when they passed through different latitudes. The results indicated significant differences between them. In stage N1, significant differences were observed between 50°N and 65°N (Figure S1, *p* < 0.05, Mann–Whitney test, *n* = 7 for Eastern migratory population, and *n* = 6 for Western migratory population, hereinafter the same); in stage S, significant differences were found in the range from 40°N to 65°N (*p* < 0.05, Mann–Whitney test, Figure S1). For the remaining stages, no significant differences were detected (*p* > 0.05, Mann–Whitney test) (Figure S1). There were no significant differences (*p* > 0.05, Mann–Whitney test) between the two populations in the following parameters: the latitude of the reached breeding sites, stopover duration, migration distance, migration duration, migration speed, number of stopover sites, arrival and departure time at the breeding sites, and arrival time at the non‐reeding sites. In stage S, the flight time of the Eastern migratory population (average 18.790 days) was significantly longer than that of the Western migratory population (average: 9.381 days). There was also a significant difference in flight speeds (V_‐Eastern_: 539.73 km/day < V_‐Western_: 955.72 km/day, *p* < 0.05, Mann–Whitney test). The single individual of the Kamchatka Peninsula was excluded from the comparison.

### 3.3 Analysis of Stopover Sites Based on the Hangzhou Bay Population

**FIGURE 2:**
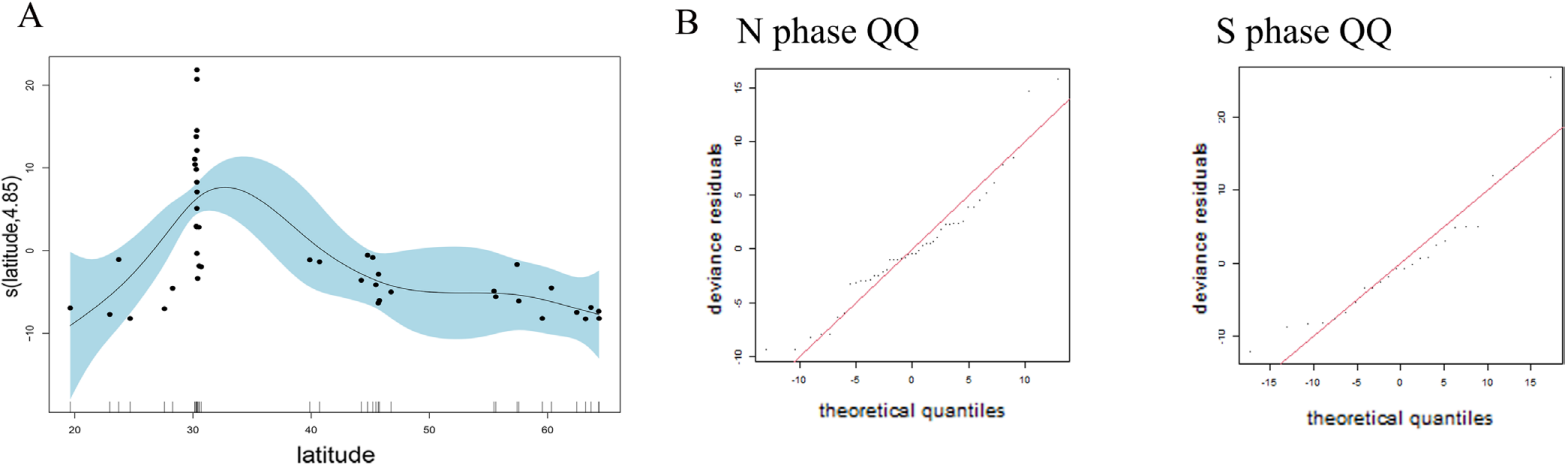
(A) The influence of latitude on the stopover duration time during the northward migration, using a generalized additive model, (B) the QQ.

During the migration of individuals from Hangzhou Bay, a total of 58 stopover events were recorded. Employing the GAM model, we discovered a significant nonlinear relationship between latitude and stopover duration at stopover sites during the northward migration phase (N1 + N2). The model could explain 51.2% of the variance and 55.1% of the deviance, suggesting that latitude significantly influenced the stopover duration. Based on the model fitting results, a peak stopover period occurred at 30°–35°N during the northward migration phase. The significance test of the smoothed term in the model (*p* < 0.05) further supported this conclusion, indicating that the latitude variable was highly significant in the model (Figure 2). We conducted model diagnostics to verify the model's plausibility and stability. The residual plots showed that the residuals were randomly distributed without a clear pattern, and the QQ plots indicated that the residuals generally followed a normal distribution (Figure 2). For stage S, latitude also had a significant nonlinear effect on the stopover duration. The model could explain about 29.3% of the variance and 32.4% of the deviance, though this effect was less significant compared to that in stage N.

Based on the above analysis, we focused on stopover sites in different regions in China. The average event of stopover and stopover duration in the East sea–South Sea coastal areas were not substantially higher than other regions (Figure S2A,C), and the average total stopover duration was significantly higher (Figure S2B). This indicates that these coastal areas are crucial stopover areas for the Whimbrel's migration (Table S2). We further analyzed the re‐utilized stopover sites in these regions. The results showed that three stopover sites were reused by individuals: Hangzhou Bay (32 events, 13 individuals), Donggang City (3 events, 2 individuals), and Panjin Wetland (5 events, 3 individuals). These three areas were utilized in both the N and S migration stages (Table S4). In Hangzhou Bay, the area with the highest activity in both the N and S phases was the mudflats east of the Hangzhou Bay Wetland Park, followed by the mudflat area near Ximen Town on the south shore of Hangzhou Bay. The overlapping area of N and S migration was 103.2 km^2^, accounting for 47.7% of the activity area during the northward migration (216.5 km^2^) and 40.6% of the activity area during the southward migration (254.2 km^2^) (Figure S3A). In the stopover site near Donggang, the stopover areas overlap between two individuals was not high, but there was an overlap between the same individual at different stages (Individual 638G06) (Figure S3B). A similar phenomenon was observed in the stopover site of two individuals in Panjin Wetland (Figure S3C).

### 3.4 Protection of Whimbrel Stopover Sites in Coastal Wetlands of China

Combined with the published data, we summarized and identified a total of 52 stopover sites for the Whimbrel in the EAAF in China. Among these, 9 were used by tracked individuals from multiple ringing sites, and 4 were used by multiple tracked individuals from the same ringing site (Table S3). At both the ringing‐site level (Hangzhou Bay and RB) and the species level (overall analysis), the stopover duration at re‐used stopover sites were significantly longer than those at single‐use stopover sites (Table S5). Notably, except for the ringed individuals from Hangzhou Bay in this study, individuals from the other four ringing sites did not utilize this wetland as a stopover site.

**FIGURE 3:**
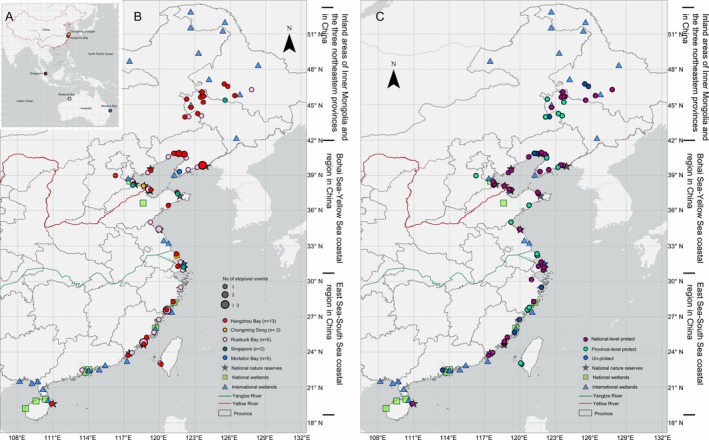
(A) The location of captured individuals; (B) the distribution of stopover sites of Whimbrels labeled in different sites; and (C) the protection status of stopover sites of Whimbrels, using the WGS84 Coordinate System. Individuals were captured in Hangzhou Bay in China (our study), Chongmingdongtan in China (Kuang et al. 2022), Moreton Bay (MB) (Kuang et al. 2020), Roebuck Bay (RB) in Australia (Kuang et al. 2020), and Singapore (Li et al. 2020).

Among the 52 stopover sites of Whimbrel in China, 31 fall within the defined national protection range and 7 within the provincial‐level protection range. In total, 73.1% of the overall area of these stopover sites is under protection (Figure 3), and 80.3% of the stopover duration occurs within the protected areas. The total stopover duration, stopover times, and average stopover duration at the national protected sites are higher than those at the provincial‐level protected and unprotected sites (Figure S4). In the East Sea‐South Sea coastal area and the Bohai Sea‐Yellow Sea coastal area, the total stopover duration at the national protected sites is 8.9 and 72.2 times higher than that at the provincial‐level protected sites respectively. This disparity is less pronounced in the inland areas of Inner Mongolia and the three northeastern provinces. In the Bohai Sea‐Yellow Sea coastal region, the stopover duration in the provincial‐level protected range is significantly shorter than that in the national protected range (*p* < 0.05, Mann–Whitney's test), indicating that the national protected areas in this region play a crucial role. Eight national nature reserves support multiple stopover events: Binzhou Shell Dike Island and Wetland, Dandong Yalujiang Wetland, Yellow River Delta, Jiuduansha Wetland, Shenhu Bay Submarine Ancient Forest Remains, Shuangtai River Mouth, Yancheng Wetland Rare Bird, and Zhangjiangkou Mangrove Nature Reserves. Only two of these reserves are located in the East Sea‐South Sea coastal area, while the remaining six belong to the Bohai Sea‐Yellow Sea coastal area.

A comparison of the three regions revealed that the proportion of protected stopover sites was lowest in the inland areas of Inner Mongolia and Northeast China, at 57.1% (8 of 14 sites), compared to 82.4% (14 of 17 sites) in the East Sea–South Sea coastal area and 72.7% (16 of 22 sites) in the Bohai Sea–Yellow Sea coastal area (Figure S4). Regarding stopover events, the total stopover duration did not differ substantially between the East Sea–South Sea and Bohai Sea–Yellow Sea coastal areas, and both regions exhibited longer total stopover duration than the inland areas of Inner Mongolia and Northeast China. Both the total number of stopover sites and the event of stopover events were higher in the Bohai Sea – Yellow Sea coastal protected areas (16 protected sites with 26 stop events) compared to those in the East Sea–South Sea coastal areas (14 protected sites with 28 stop events) (Table 2; Figure S4A,C).

**TABLE 2:** Comparison between protected and unprotected stopovers. The duration time was represented by the median value.

Areas	Parameters	East Sea‐South Sea coastal region	Bohai Sea‐Yellow Sea coastal region	Inner Mongolia and the three northeastern provinces (China)
National‐level protected	Event	14	24	8
	Event percentage of the whole events in China	58.3%	70.5%	47.1%
	Total duration (days)	428.9	418.9	53.8
	Duration percentage of the whole duration days in China	81.6%	74.8%	38.2%
	Median duration (days)	10.0	13.1	6.8
	Range (days)	2.00–22.98	4.00–39.00	3.14–9.99
Provincial‐level protected	Event	4	2	3
	Event percentage of the whole events in China	16.7%	5.9%	17.6%
	Total duration (days)	48.0	5.8	29.4
	Duration percentage of the whole duration days in China	9.1%	1.0%	20.9%
	Median duration (days)	9.5	2.9	11.0
	Range (days)	2.00–27.00	2.84–3.00	5.36–13.00
Unprotected	Event	6	8	6
	Event percentage of the whole events in China	25.0%	23.5%	35.3%
	Total duration (days)	49.0	134.9	57.7
	Duration percentage of the whole duration days in China	9.3%	24.1%	40.9%
	Median duration (days)	4.2	11.3	7.9
	Range (days)	2.00–24.00	3.97–41.00	3.16–23.00
*p* values of duration time with Mann–Whitney *U* test	National versus provincial	*p* = 0.48	*p* < 0.05	*p* = 1.00
	National versus unprotected	*p* = 0.36	*p* = 0.68	*p* = 0.42
	Provincial versus unprotected	*p* = 0.42	*p* < 0.05	*p* = 0.89

## 4 Discussion

Here, we analyzed the migratory pathways of the Whimbrel ringed in Hangzhou Bay using satellite tracking data, and evaluated the protection status of their stopover sites by integrating published data. Our findings indicated that after traversing the northeastern plains of China, the species established two distinct migratory routes leading to different breeding regions. No significant differences were observed in the utilization of stopover sites across various migratory phases; individuals demonstrated a high fidelity to Hangzhou Bay, with all individuals repeatedly using it as a stopover site during both northward and southward migrations. By combining our tracking data with those from other studies, we found that nearly three‐quarters of the Whimbrel's stopover sites in China's coastal wetlands are covered by national and provincial protection schemes. Notable disparities exist in the protection status of stopover events among different regions. Based on the analysis of stopover sites and total stopover duration, the inland areas of Inner Mongolia and the three northeastern provinces currently represent the weakest links in terms of protection levels, highlighting a pressing need for enhanced conservation management in these critical regions.

### 4.1 Co‐Existence of Two Subspecies of Whimbrel in the EAAF Pathway

According to IOC v14.2 (Gill et al. 2024), the nominate subspecies *Numenius phaeopus* comprises five subspecies. Among them, *N. p. variegatus* and *N. p. rogachevae* are associated with the EAAF migration. It is thought that only *N. p. variegatus* inhabits Southeast Asia and Australia within the EAAF. In contrast, the *N. p. rogachevae* subspecies spends its non‐breeding season in West India and eastern Africa (Gill et al. 2024; Skeel and Mallory 2020), migrating through the Central Asian flyway to breed in central Siberia (Li et al. 2020). However, based on morphological and migratory tracking analyses, Kuang et al. (2022) demonstrated that *N. p. rogachevae* also utilizes the EAAF for migration. Similar shared migratory corridors among different subspecies have been observed in other species, such as the Black‐tailed Godwit ( *Limosa limosa* ) (Zhu et al. 2023) and Dunlin (Lagassé et al. 2022). The eastern subspecies (*melanuroides*) and the Bohai subspecies (*bohaii*) of Black‐tailed Godwit overlap in multiple stopover and non‐breeding areas within the EAAF (Zhu et al. 2023). For Dunlin, although their four subspecies exhibit spatiotemporal differences in migration pathways, they proportionally utilize several identical regions, including the Kuril Islands and the Yellow Sea area (Lagassé et al. 2022). These findings suggest that reproductive isolation is a primary driver of species differentiation (Sobel et al. 2010). In shorebirds, breeding site differences play a crucial role in this process. For instance, the breeding areas of Black‐tailed Godwit subspecies vary significantly: the Bohai subspecies breeds near the Arctic Circle in the Russian Far East (61°–68°N), whereas the eastern subspecies nests in the temperate steppe lakes of Asia (42°–52°N) (Zhu et al. 2021).

The breeding longitudes of the Eastern and Kamchatka Peninsula migrants (80°–120°E) and Western migrants (130°–180°E) among the Hangzhou Bay‐ringed individuals confirm the presence of two subspecies of *N. p. rogachevae* along the EAAF migratory pathway. Analysis of Hangzhou Bay‐tracked individuals revealed that the migration routes of these two groups were identical within China's coastal wetlands. The routes diverged after stopovers in the grassland wetlands of the three northeastern provinces (north of 50°N), leading to different breeding areas. They also shared key stopover locations, and the western population exhibited prolonged stopovers at Hangzhou Bay during the S stage migration (likely for extended energy replenishment), while maintaining similar migration speeds to the eastern population. However, the genetic characterization of the subspecies affiliation for these migrating individuals remains unaddressed and requires further investigation in future studies.

### 4.2 Protected Status of Shorebirds' Migration Pathway

In the intricate migratory network of birds, stopover sites serve as crucial nodes in this dynamic system. Shorebirds rely on these sites to feed and accumulate energy, which is essential for meeting the demands of migratory flights and breeding activities. Consequently, the energy‐rich conditions of stopover sites play a vital role in both the migratory and breeding behaviors of shorebirds. Research in the coastal wetlands of the Yalu River has demonstrated that the quality and quantity of food available at stopover sites are the primary determinants of shorebirds abundance (Zhang et al. 2018). Along the Yellow Sea coast, the total area of tidal flats at 14 stopover sites has decreased by 35.6%, leading to a 7.8% decline in the number of shorebirds. This reduction has intensified competition for space and food among shorebirds at these stopover sites (Wang et al. 2022). Long‐term monitoring data from Bohai Bay by Liu et al. (2022) revealed that the loss of stopover sites forces migratory waterbirds to prolong their stay at remaining sites for energy replenishment, leading to increased bird density at these locations. Consequently, the remaining stopovers become stages where density‐dependent effects are most pronounced along the migration route. Piersma et al. (2016) found that the habitat quality of the Bohai Sea–Yellow Sea wetland was correlated differently with various shorebird species. While it showed no significant relationship with some species, it had a strong positive correlation with the summer survival of the Red Knot ( *Calidris canutus piersmai* ), Great Knot ( *Calidris tenuirostris* ), and the Bar‐tailed Godwit ( *Limosa lapponica menzbieri* ) (Piersma et al. 2016). A study on Great Knot indicated that the loss and/or degradation of migratory stopover sites can lead to changes in the migratory phenotype composition at the population level. Specifically, individuals with later migration schedules and lower energy reserves are less likely to survive (Peng et al. 2023). Collectively, these studies underscore the critical importance of stopover sites for migratory birds.

In migratory networks, the loss of key stopover sites can trigger the collapse of the entire migratory system. Moreover, insufficient understanding of the timing, intensity, and duration of stopover site utilization can impede the development of effective conservation strategies for migratory birds. Shorebirds often exhibit high site fidelity to both breeding and stopover locations, as seen in species like the Marbled Godwit ( *Limosa fedoa* ) and Willet ( *Tringa semipalmata* ) (Sandercock and Gratto‐Trevor 2023). Wang et al. (2022) observed in the Bohai Sea–Yellow Sea wetlands that while the number of migratory birds declined with decreasing habitat quality, the avian community composition remained relatively stable. This suggests that habitat loss at one stopover site cannot be easily compensated by protecting other sites, underscoring the necessity of safeguarding numerous existing critical stopover areas for migratory bird conservation. In China's coastal seashore wetlands, there are 16 Ramsar International Important Wetlands, 9 National Important Wetlands, and 66 Important Bird Migration Areas (Conklin et al. 2014). Sites such as the Chongming Dongtan, Dongtai Tiaozini, Rudong Wetlands, Nanbao Mudflats, Dandong Yalu River Estuary Wetlands, and inland Xingkai Lake in Heilongjiang serve as crucial stopovers for migratory shorebirds, playing an irreplaceable role in maintaining biodiversity (Wei et al. 2023).

The Whimbrel studied here represents a significant population within the EAAF, making it a key species for shorebird migration conservation. Our study of individuals captured in Hangzhou Bay determined that the peak stopover period occurred between 30° and 35° N during the northward migration stage, which aligns with An et al. (2024)'s finding of highest migration route consistency at 35°N based on RB, MB, and CMDT individuals (using 10° intervals), demonstrating good consistency between the two studies. Compared with more than 10 repeat‐used stopover sites in An et al. (2024), widespread in coastal China, our study just determined two in the Yellow Sea coast, and only along the East China Sea coast (Hangzhou Bay). This might suggest that An et al. (2024) used individuals that were tracked for more than one year. However, the highutilization of Hangzhou Bay revealed in our study provides critical complementary data to existing knowledge on Whimbrel migration ecology. Based on tracking data from this study and published sources (*n* = 27 in all), our synthesis shows 73.1% of documented stopover sites and 80.3% of stopover time occur within protected areas. However, these findings should be interpreted as minimum estimates, as undiscovered stopover habitats may exist outside current tracking coverage. The effectiveness of different protection levels varies. National‐level nature reserves, national‐level wetland parks, and national‐level forest parks, which typically cover larger contiguous areas, contribute most significantly to protecting the species' migration in China, covering 59.6% of stopover sites and 73.3% of stopover duration time. Provincial‐level nature reserves, some of which are fragmented and small in coverage, help fill gaps along migration routes but require enhanced protection efforts. Management and conservation investment vary across protection levels in China's protected areas, with national‐level reserves receiving stronger central support for large‐scale ecosystems, while provincial‐level reserves focus on fragmented regional habitats. Overall, stopover site protection in the inland areas of Inner Mongolia and the three northeastern provinces lags behind that in the Bohai Sea–Yellow Sea and East Sea –South Sea coastal regions. The lower proportion of stopover duration time within protected areas in these inland regions is mainly due to some critical areas being excluded from protection. However, in this region, some repeat‐used stopover sites were also determined, e.g., Baicheng in our data. Given that this region lies in the latter part of the species' migration, it significantly impacts reproductive success and warrants heightened attention in future conservation efforts.

Existing research has documented preliminary assessments of wetland conservation status in China relative to migratory bird protection. Based on waterbird survey data from bird‐themed websites, survey reports, and literature, 58.46% (38 out of 65) of the identified priority coastal waterbird conservation areas in China have protection gaps (Duan et al. 2021). In the Yellow River Basin and Northwest China, this proportion is 42.55% (20 out of 47) (Duan et al. 2021) and 35.42% (17 out of 48) (Li et al. 2020). An analysis of 33 complete spring migration routes of Anatidae in China revealed that the existing protection network covers only 15.63% (342,757 ha) of the stopover sites (Lei et al. 2019). Although wetlands in the Yellow River Basin, which are dominated by Charadriiformes and Anseriformes waterbirds, had a protection rate of 65% by the end of 2020 (Sun et al. 2020; Wang et al. 2024) analysis of the Spoonbill ( *Platalea leucorodia* ) and Oriental White Stork ( *Ciconia boyciana* ), using 50% of their home range as a reference, showed that the coverage rate of protected areas was only 1.6% and 0 respectively (Wang et al. 2020). Compared with these statistics, the protection of shorebirds in China's coastal regions has benefited from the establishment of multiple protected areas. However, to achieve the goal of “by 2030, 90% of the critical habitats of migratory birds in migration corridors will be effectively protected, forming a more comprehensive national migratory bird migration corridor protection network,” it is crucial to monitor and safeguard important waterbird stopover sites currently outside protected‐area boundaries. China's ecological red line system offers a promising solution for achieving this objective (Choi et al. 2022).

Previous studies in Hangzhou Bay predominantly focused on waterbird diversity, yet lacked in‐depth exploration of their functions and roles within migration corridors. Tracking data of shorebirds, exemplified by the Whimbrel, reveal that these birds consistently utilize the wetlands of Hangzhou Bay as a stopover site. The Whimbrel exhibits a high degree of site fidelity to Hangzhou Bay, although the population also briefly uses two other nearby stopover sites: Yangpu District in Shanghai and Rudong Wetland in Jiangsu. In Hangzhou Bay, the activity range of the Whimbrel population is notably concentrated, with significant overlap in individual activity areas—a characteristic distinct from other re‐used stopover sites. The core activity range of the Whimbrel lies outside the Hangzhou Bay National Wetland Park. Long‐term synchronized surveys of wintering waterbirds in Zhejiang Province by our team have shown that a large number of wintering waterbirds gather at high‐tide points within this core activity area (unpublished data). Collectively, these findings underscore the critical importance of Hangzhou Bay coastal wetlands for the migration of this population. Research has indicated that the fidelity of different Whimbrel populations to their stopover sites influences population size, with high densities often indicative of intense intraspecific competition. As of March 2025, this area has not been designated as a national‐level important wetland. Currently, the existing national wetland park has a limited protection area, highlighting an urgent need for increased attention and enhanced conservation efforts.

## 5 Conclusions

In this study, we analyzed the migration routes, stopover sites, and stopover protected status of the Whimbrel. The findings revealed the presence of two subspecies at EAAF stopover sites in Hangzhou Bay. Nationally, 73.1% of stopover sites were within protected areas; however, in the inland regions of Inner Mongolia and Northeast China, 42.9% of stopover sites remained unprotected. These results underscore the urgent need to focus on unprotected areas to enhance the conservation of shorebirds within their migration corridors.

## Author Contributions

**Ke He:** conceptualization (equal), data curation (equal), software (equal), supervision (equal), visualization (equal), writing – original draft (equal), writing – review and editing (equal). **Zhen Xian Zhu:** data curation (equal). **Tinghao Jin:** conceptualization (equal), data curation (equal), software (equal), visualization (equal), writing – original draft (equal). **Xilai Zhou:** data curation (equal), software (equal). **Jun Yan Feng:** data curation (equal), visualization (supporting). **Lingling Wei:** software (supporting). **Baoquan Liu:** conceptualization (equal), funding acquisition (equal), supervision (equal), writing – review and editing (equal).

## Conflicts of Interest

The authors declare no conflicts of interest.

## Supporting information


**Figure S1:** Comparison of the longitudes of different breeding population groups when passing through the fixed latitude. (A) N1 phase (Eastern migratory population *n* = 7, Eastern migratory population *n* = 6), (B) S phase (Eastern migratory population *n* = 7, Eastern migratory population *n* = 6), and (C) N2 phase (Eastern migratory population *n* = 3, Eastern migratory population *n* = 4). *p < 0.05, **p < 0.01.
**Figure S2:** (A) The average duration of stopovers in different regions, (B) the average total stopover duration in different regions, and (C) the average single‐stopover duration in different regions. The average time of stopovers is calculated by dividing the total numbers of stopovers by the number of individuals‐times in the monitoring phase. The average total stopover duration is obtained by dividing the total duration of stopover days by the number of individuals‐times in the monitoring phase. The average duration of a single stopover is calculated by dividing the total number of stopover days by the number of stopovers.
**Figure S3:** The activity zone of (A) Hanghzou Bay, (B) Dongdang city, and (C) Panjin Wetland.
**Figure S4:** Protected status of (A) stopover sites, (B) overall stopover duration, (C) stopover events, and (D) average duration of a single stopover of Whimbrels in different regions of China.

**Table S1:** Tracking information of Whimbrel in this study.
**Table S2:** Information of stopover sites of the labeled individuals in Hangzhou Bay in this study.
**Table S3:** Data of Whimbrel's stopover sites from literatures and our study.
**Table S4:** Information on the re‐used stopover sites by the Whimbrel along the coast of China.
**Table S5:** Comparison of the duration time in re‐used stopover sites and non‐repeatedly used stopover sites.

## Data Availability

All the required data are uploaded as Supporting Information.
