# Supplementary material for: Migration Ecology and Protection of Stopover Sites of the Whimbrels Along China's Coastal
Source: Ecol Evol. 2025 Aug 8;15(8):e71890. doi: 10.1002/ece3.71890 (PMC12333071; doi:10.1002/ece3.71890)
Supplement: Supplementary file 1 — Figure S1: Comparison of the longitudes of different breeding population groups when passing through the fixed latitude. (A) N1 phase (Eastern migratory population n = 7, Eastern migratory population n = 6), (B) S phase (Eastern migratory population n = 7, Eastern migratory population n = 6), and (C) N2 phase (Eastern migratory population n = 3, Eastern migratory population n = 4). *p < 0.05, **p < 0.01. Figure S2: (A) The average duration of stopovers in different regions, (B) the average total stopover duration in different regions, and (C) the average single‐stopover duration in different regions. The average time of stopovers is calculated by dividing the total numbers of stopovers by the number of individuals‐times in the monitoring phase. The average total stopover duration is obtained by dividing the total duration of stopover days by the number of individuals‐times in the monitoring phase. The average duration of a single stopover is calculated by dividing the total number of stopover days by the number of stopovers. Figure S3: The activity zone of (A) Hanghzou Bay, (B) Dongdang city, and (C) Panjin Wetland. Figure S4: Protected status of (A) stopover sites, (B) overall stopover duration, (C) stopover events, and (D) average duration of a single stopover of Whimbrels in different regions of China. [file ECE3-15-e71890-s001.docx]

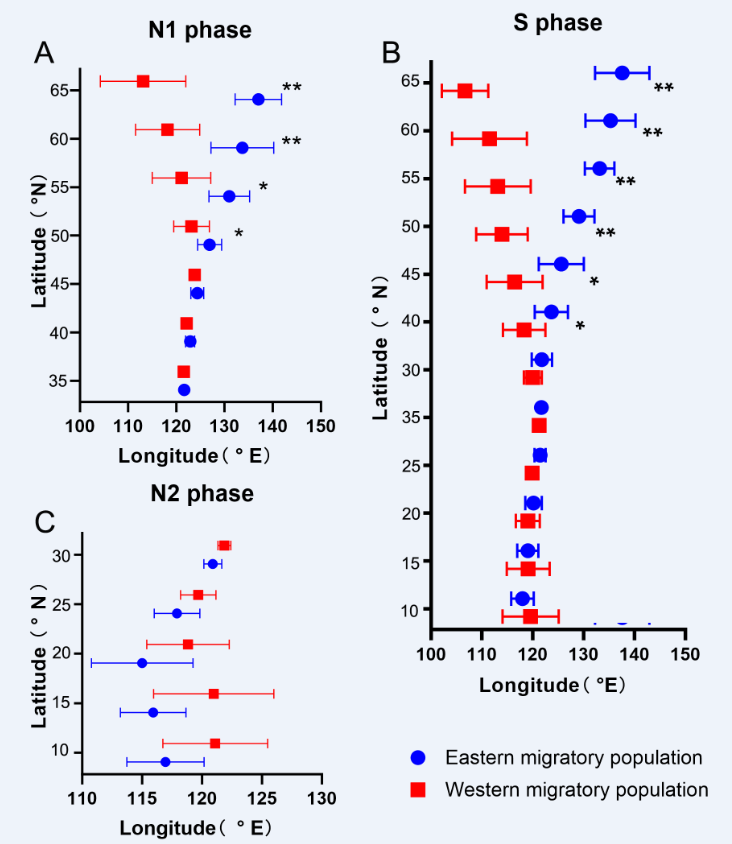


Fig S1 Comparison of the longitudes of different breeding population groups when passing through the fixed latitude. (A) N1 phase (Eastern migratory population n=7, Eastern migratory population n=6), (B) S phase (Eastern migratory population n=7, Eastern migratory population n=6) , and (C) N2 phase (Eastern migratory population n=3, Eastern migratory population n=4). * *P* <0.05, ** *P*<0.01.


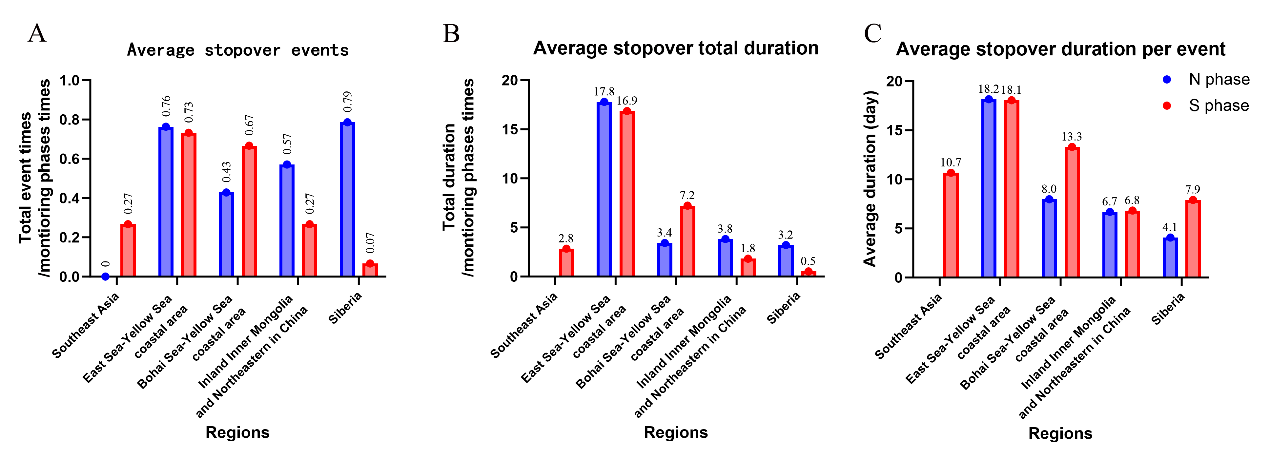


Fig S2 (A) The average duration of stopovers in different regions, (B) the average total stopover duration in different regions, and (C) the average single-stopover duration in different regions. The average time of stopovers is calculated by dividing the total numbers of stopovers by the number of individuals-times in the monitoring phase. The average total stopover duration is obtained by dividing the total duration of stopover days by the number of individuals-times in the monitoring phase. The average duration of a single stopover is calculated by dividing the total number of stopover days by the number of stopovers.


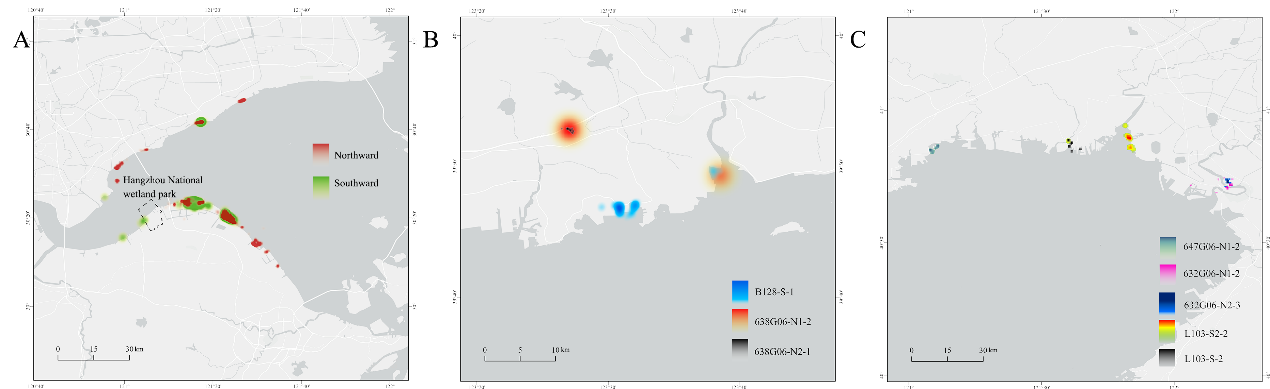


Fig S3 The activity zone of (A) Hanghzou Bay, (B) Dongdang city, and (C) Panjin Wetland.


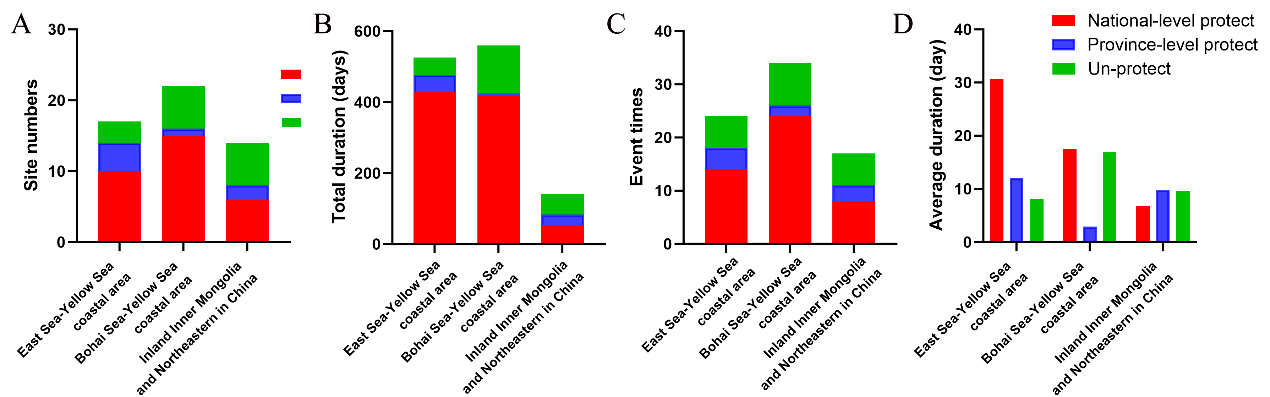


Fig S4 Protected status of (A) stopover sites, (B) overall stopover duration, (C) stopover events, and (D) average duration of a single stopover of Whimbrels in different regions of China.
